# Earthworm occurrence dataset extracted from Russian-language literature

**DOI:** 10.3897/BDJ.12.e130897

**Published:** 2024-12-16

**Authors:** Maxim Shashkov, Natalya Ivanova, Sergey Ermolov

**Affiliations:** 1 Institute of Mathematical Problems of Biology RAS – the Branch of Keldysh Institute of Applied Mathematics of Russian Academy of Sciences, Pushchino, Russia Institute of Mathematical Problems of Biology RAS – the Branch of Keldysh Institute of Applied Mathematics of Russian Academy of Sciences Pushchino Russia; 2 Center for Forest Ecology and Productivity RAS, Moscow, Russia Center for Forest Ecology and Productivity RAS Moscow Russia

**Keywords:** data mining, data mobilisation, grey literature, Northern Eurasia, taxonomic checklists

## Abstract

**Background:**

Earthworms are one of the most important components of temperate ecosystems and groups of soil animals globally, but data on their distribution around the world are still incomplete and uneven. Northern Eurasia is a region for which available data on earthworm distribution is extremely poor. At the same time, generations of Soviet and Russian researchers have performed extensive research and accumulated a large amount of data on the distribution of earthworms in this vast region. Most of this information was published in Russian, not digitised and, therefore, hardly accessible to researchers. Our dataset fills this gap and provides occurrence data extracted from 159 literature sources in Russian published between 1868 and 2022. The dataset contains 5,304 occurrences of 109 species and 14 subspecies. The collected data cover the territory of 27 countries.

**New information:**

The dataset extends the data available through GBIF.org on earthworm distribution in Russia, Belarus, and Ukraine. For Transcaucasia and Central Asia countries, our resource has become the main GBIF source on earthworm diversity distribution. On a global scale, our dataset contributes to filling gaps in biodiversity, which facilitates more accurate estimates of the response of biodiversity to global climate change as well as the spreading of invasive species.

## Introduction

Earthworms, a key group of organisms in terrestrial ecosystems, belong to the families of the order Crassiclitellata, also known as the "Megadrili" group (Lavelle 2014), yet their global distribution remains under-represented in digital resources. Dozens of earthworm species, both widespread and endemic, inhabit the territory of Northern Eurasia (Perel 1979), but extremely poor data on them are available through global biodiversity repositories (Cameron et al. 2018). Since the end of the 19^th^ century, a number of generations of Russian and Soviet researchers have studied the diversity of earthworms in Northern Eurasia. A large amount of data on earthworm communities has been collected from the European part of Russia (Petrov 1947, Gavrilov and Perel 1958, Ustinov 1962, Sharova 1970, Penev et al. 1994, Geraskina 2009, Gruntal 2010, Rybalov and Kamaev 2011), the Russian Caucasus (Vsevolodova-Perel 2003, Rapoport and Komarov 2013, Geraskina 2016b), Ural (Perel 1967, Vorobeichik 1998, Ermakov and Golovanova 2010, Geraskina 2016a), Altai (Golovanova et al. 2015), Siberia (Byzova and Chadaeva 1965, Ermolov 2019), Russian Far East (Ganin 1994, Berman et al. 2001), including permafrost areas (Chernov 1961, Ubugunova et al. 2007, Makarova and Kolesnikova 2019). Soviet scientists furthermore have collected data from Moldova (Malevich 1955, Perel 1962, Striganova 1968), Belarus (Malevich 1953), Ukraine (Malevich 1954, Malevich et al. 1954), Azerbaijan, Georgia, Armenia (Bababekova 1965, Kvavadze 1973), Turkmenistan, Uzbekistan, Tajikistan, Kazakhstan, Kyrgyzstan, and Mongolia (Malevich 1945, Malevich 1949, Malevich 1959, Valiakhmedov and Perel 1961, Perel 1971). Most of these data have been published in grey literature, which is now only available as hard copies in a few libraries. Until recently, most of these publications remained largely undigitised and some are probably irretrievably lost.

In recent years, some local datasets on earthworm diversity in Russia have been published through the GBIF portal. These data present results of long-term surveys in forests of the Central Urals (Vorobeichik et al. 2021) and old-growth broad-leaved forests of European Russia (Shashkov et al. 2021), as well as occurrences collected from different places (Shashkov et al. 2019, Konakova et al. 2020, Nakonechniy and Golovanova 2020). Overall, these data are fragmented and have many gaps in both space and time. For other former USSR countries, earthworm data are derived mainly from global resources (The International Barcode of Life Consortium 2016, Timm and Abarenkov 2017), excluding Ukraine and Belarus, where occurrence data collected by local researchers were published (Harbar et al. 2021, Shekhovtsov et al. 2023). For Transcaucasia and Central Asia countries, only a few records were available through GBIF. For example, from Turkmenistan and Uzbekistan, three lumbricid occurrences were known from each country, two occurrences from Armenia, and six ones from Azerbaijan.

Thus, the occurrence data available through the GBIF portal does not reflect the actual distribution of earthworm species. This data deficiency may lead to biased estimates of diversity and inadequate assessments of range dynamics, as well as misleading distribution models of earthworm species at global and regional scales. Digitisation and publication of data accumulated in the Soviet and Russian scientific literature will significantly increase the completeness and spatial coverage of the available data on earthworms. In addition, this work is important by itself for the preservation of the scientific heritage accumulated by Soviet and Russian scientists.

Here, we provide the occurrence dataset (Shashkov et al. 2023) that is based on the manually extracted locations of earthworms from available literature published in Russian, spanning from the late Russian Empire to the present day.

## Sampling methods

### Sampling description

**Literature mining**

To compile a list of earthworm publications, we surveyed publications by major Soviet and modern (since the 1990s) Russian soil zoologists and their successors. We also screened the tables of content of Russian peer-reviewed journals available through the Russian Scientific Electronic Library eLibrary:

- Russian Journal of Zoology [Zoologicheskij zhurnal] ISSN 0044-5134;
- Eurasian Soil Science [Pochvovedenie] ISSN 0032-180X;
- Russian Journal of Forest Science [Lesovedenie] ISSN 0024-1148;
- Biology Bulletin Reviews [Uspehi sovremennoj biologii] ISSN 0042-1324;
- Biology Bulletin [Izvestija rossijskoj akademii nauk. Serija biologicheskaja] ISSN 1026-3470;
- Russian Journal of Ecology [Jekologija] ISSN 0367-0597;
- Proceedings of the Russian Academy of Sciences [Doklady Akademii Nauk] ISSN 0002-3264;
- Bulletin of Moscow Society of Naturalists [Bjulleten' Moskovskogo obshhestva ispytatelej prirody. Otdel biologicheskij] ISSN 0027-1403.

Finally, we surveyed the abstracts of the All-Russian Soil Zoology Conference and other relevant topical conferences, as well as other conference abstracts available through the eLibrary scientific digital library.

**Figure 1.**
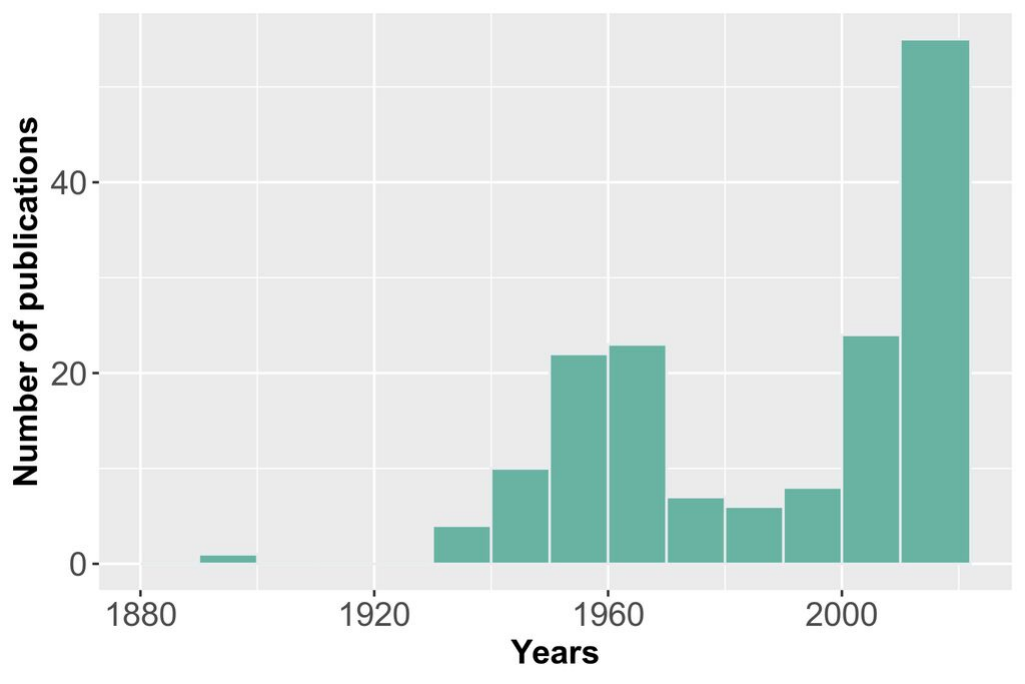
Distribution of the publications by year.

Therefore, about 1,000 publications were screened for earthworm occurrences. For further processing, 330 publications were selected, based on titles, of which 219 full-text publications were retrieved from libraries. Amongst them, only 159 sources were included in the dataset published through GBIF. The remaining publications do not contain location data, even at the country level or the information has already been entered into the dataset from other papers. Most of the processed sources were peer-reviewed articles (Table 1). The oldest article included in the dataset was published in 1899 (Vysotsky 1899). Most of the publications belong to the period from the 1950s to the present (Fig. 1).

Most of the processed publications were not accessible via the Internet and do not have global identifiers (DOIs or eLibrary IDs). In general, Russian-language scientific journals are little known to the international scientific community, and some journals have no International Standard Serial Number (ISSN) or are no longer published. Therefore, we have compiled a detailed table with the bibliographic data of all publications included in the GBIF dataset. The structure of this table is similar to the Literature References Darwin Core Extension (Anonymous 2023), but there are a number of additions. A full list of publications included in the dataset is available through Zenodo (Ivanova et al. 2023).

**Data digitisation**

**Figure 2.**
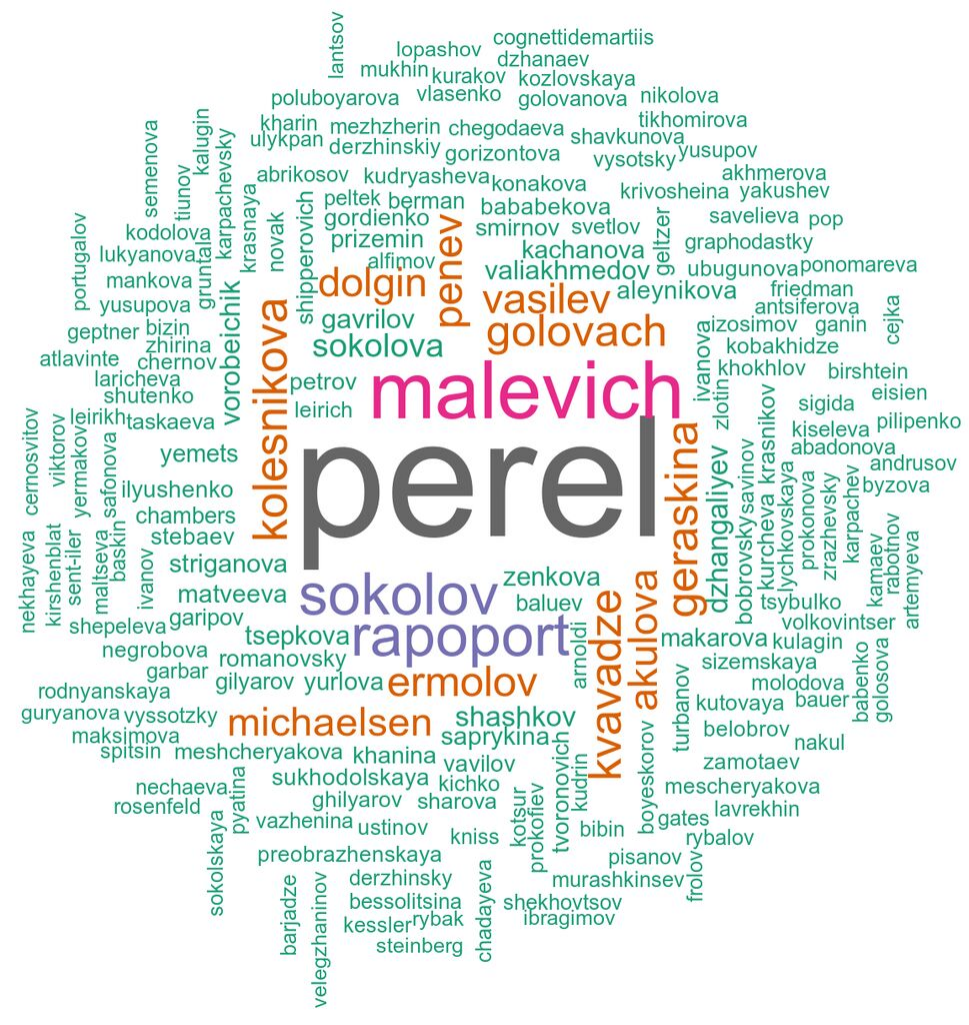
A WorldCloud chart of the frequency of occurrences per collector in the dataset.

Occurrence data were extracted and structured according to the Darwin Core standard (Wieczorek et al. 2012). During the data digitisation process, we have tried to provide as much primary information as possible. For each occurrence, the relative abundance (dwc:organismQuantity and dwc:organismQuantityType), number of individuals (dwc:individualCount), and the life stage (dwc:lifeStage) were recorded if such data were provided. Survey data were digitised by keeping the data structure, using dwc:eventID. We kept the original habitat categories or descriptions (see dwc:habitat) in Russian because the authors used different approaches and appropriate translation was not always possible. Each occurrence has a reference to the source. We provided the date of collection in the dwc:verbatimEventDate field with the detail that was available in the text. If the authors did not provide the date of the survey, we specified the year of publication in the dwc:eventID field and added a comment in the dwc:eventRemarks field. In the dwc:recordedBy field, the author(s) of the publication was/were mentioned. The primary authors of the occurrence were also noted here if this information was specified in the text. The identifier (dwc:IdentifiedBy) was recorded only if this person was mentioned in the text. Overall, the data we collected adequately reflects the efforts of researchers to collect earthworms (Fig. 2). According to the update of the Darwin Core standard, the basis of record (dwc:basisOfRecord) of a literature-based occurrence should be "MaterialCitation", but the actual version of IPT does not provide this value within the controlled vocabulary. Thus, we specified "HumanObservation" throughout the dataset, although some of the sampled specimens might have been stored in collection.

During the data digitisation process, we recorded the original scientific names in dwc:identificationRemarks (dwc:verbatimIdentification is not supported by the current IPT version – 3.0.4). We kept the original spelling of the scientific names, except for obvious typos and fluctuations in the use of punctuation marks. The dwc:scientificName field represents the taxonomic names from the GBIF Backbone Taxonomy checklist (GBIF Secretariat 2011) corresponding to the initial ones. Finally, scientific names were matched with the actual global checklist of earthworms (Misirlioǧlu et al. 2023), the result was recorded in the dwc:acceptedNameUsage field, and the dwc:acceptedNameUsageID field contains codes from the GBIF Backbone Taxonomy.

**Georeferencing.** We recorded verbatim location in the dwc:verbatimLocality field (in Russian). After this, we specified the country according to the United Nations political boundaries and state/provinces, if possible. For some records, we could not unambiguously identify the country, mainly because the collections were made during the Soviet Union and the locality description was not accurate enough to determine in which country it is located now.

Only 310 occurrences contained the geographic coordinates of locations provided by the authors. We converted these coordinates to decimal degrees (if necessary) and checked if they match the location description. The obviously wrong coordinates were corrected according to the description of the locality. The original coordinates were also included in the GBIF dataset in the dwc:verbatimCoordinates field. The remaining occurrences were georeferenced manually using the point-radius method (Wieczorek et al. 2010). According to this approach, each locality is described as a circle, with a point to mark the position most closely described by the locality description and a radius to describe the maximum distance from that point within which the locality is expected to occur. To find locality by text description, we used online maps (Google Maps, Yandex Maps, and OpenStreetMaps) as well as topographic maps, corresponding to the period of data collection. At the same time, 1,406 (26.5%) occurrences could not be geolocated because they did not have a detailed description of their locations.

**Figure 3.**
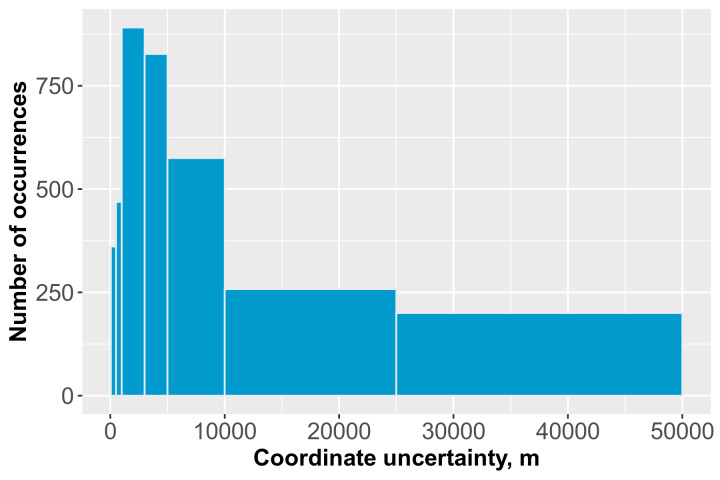
Coordinate uncertainty in metres for all georeference occurences.

For each geo-referenced occurrence (manually georeferenced and author's coordinates), we evaluated the associated uncertainty (radius). For the coordinates obtained using a GPS navigator, we specified it to be 50 m because none of the authors provided detailed information about the protocol for determining the coordinates. For the other occurrences, the uncertainty was determined expertly, taking into account the specificity of the locality description and the accuracy of the sources used to determine coordinates. If we could estimate the uncertainty radius as more than 50 km, we did not geolocate the record. The exception was the article by I.I. Malevich (Malevich 1954), where the locations were extracted from the coarse-scale schematic map rather than textual descriptions. Therefore, the average coordinate uncertainty in the dataset was 6,632.2 m, while for 76.5% of georeferenced occurrences, the coordinate uncertainty was less than 10,000 m; for 56.4% of georeferenced occurrences, it was less than 5,000 m (Fig. 3).

### Quality control

To control the quality of the data, we followed basic principles (Chapman 2005). The most difficult process was the verification of taxonomic data extracted from literature. To meet this challenge, we have published three checklist datasets based on inventories of T.S. Perel through the GBIF Portal (Shashkov 2023a, Shashkov 2023b, Shashkov 2023c) and requested the integration of them into the GBIF Backbone Taxonomy. Given that one of these lists all possible synonyms, it provides an opportunity to publish data in accordance with the authors' chosen taxonomy.

Despite this, in some cases, we had to draw on additional literature (Anonymous 1973b, Reynolds 2010, Blakemore 2013, Raxmatullayev 2023) to clarify to which species from the GBIF Backbone Taxonomy a particular occurrence corresponds. Amongst them were: *Allobophorakobachidzei* Malevich (= *Aporrectodeadubiosa* (Örley, 1881)), *Allobophoratugida* (Ros.) (= *Aporrectodeatuberculata* (Eisen, 1874)), *Allolobophoraacystis* Mich. (= Eisenianordenskioldisubsp.pallida (Malevic, 1956)), *Allolobophoracavatica* Michaelsen, 1910 (= *Aporrectodeajassyensis* (Michaelsen, 1891)), *Allolobophorakazanensis* (= *Pereliakaznakovi* (Michaelsen, 1910)), *Bimastusconstrictor* (Ros.) (= *Bimastosrubidus* (Savigny, 1826)), *Dendrobaenacernosvitovianus*, and *Eophilacernosvitoviana* (= *Helodriluscernosvitovianus* (Zicsi, 1967)), *Dendrobaenaintermedia* (= Dendrobaenaschmidtisubsp.tellermanica Perel, 1966), *Eisenialeoni* (Mich.) (= *Allolobophoraleoni* Michaelsen, 1891), *Eisenianordenskioldisibirica* (= *Eiseniasibirica* Perel & Graphodatsky, 1984). Several subspecies mentioned in the source literature were not found in the GBIF Backbone Taxonomy: Dendrobaenahandlirschivar.rhenani, *Dendrobaenaplatyuramontana*, HelodrilushandlirschiRosavar.rhenani Bretsch., Helodrilusschelkovnikovivar.veliensis nov., and HelodrilusschmidtiMichvar.violacea nov. The taxonomical status of species *Archaeodriluscavaticus* Czerniavsky, 1881, is specified as "unclear" in literature, but in the GBIF Backbone Taxonomy, it is recorded as accepted. In the course of taxonomic name verification, all formas and varieties were considered subspecies.

We have revealed many inconsistencies in the current version of the GBIF Backbone Taxonomy. The main resource on earthworm taxonomy is the World Register of Marine Species (WoRMS) (Anonymous 2024), which is partly outdated and often incomplete. Since the current version of the GBIF Backbone Taxonomy cannot be considered a reliable source of Megadrili taxonomy, we used "Earthworms (Clitellata, Megadrili) of the World: an updated checklist of valid species and families, with notes on their distribution" (Misirlioǧlu et al. 2023) for taxonomy unification. We placed the names from this checklist in the dwc:acceptedNameUsage field and the corresponding codes from the GBIF backbone in the dwc:acceptedNameUsageID field. The taxonomic status of the accepted name against the GBIF Backbone Taxonomy was specified in the dwc:taxonRemarks when it differed from the GBIF checklist. Although for most records, scientific names were the same, otherwise, they indicated either "Alternative ACCEPTED" or "Backbone synonym is ACCEPTED", when a scientific name from the Megadrili checklist is considered "accepted" in the GBIF Backbone, but differs and is considered "synonym", respectively. Besides, we revealed four species scientific names from the Megadrigi checklist that are not listed in the GBIF Backbone Taxonomy: *Dendrobaenatellermanica* Perel, 1966, *Eiseniapallida* Malevič, 1956, *Eiseniabashkirica* (Malevič, 1950), and *Cernosvitoviasturanyi* Rosa, 1895; hence, for occurrences with these accepted scientific names, the field dwc:acceptedNameUsageID is blank. In some cases, different spellings of the same species within the GBIF Backbone are both recorded as distinct accepted species: *Dendrobaena mariupoliensis* and *Dendrobaena mariupolienis* Wyssotzky, 1898.

## Geographic coverage

### Description

**Figure 4.**
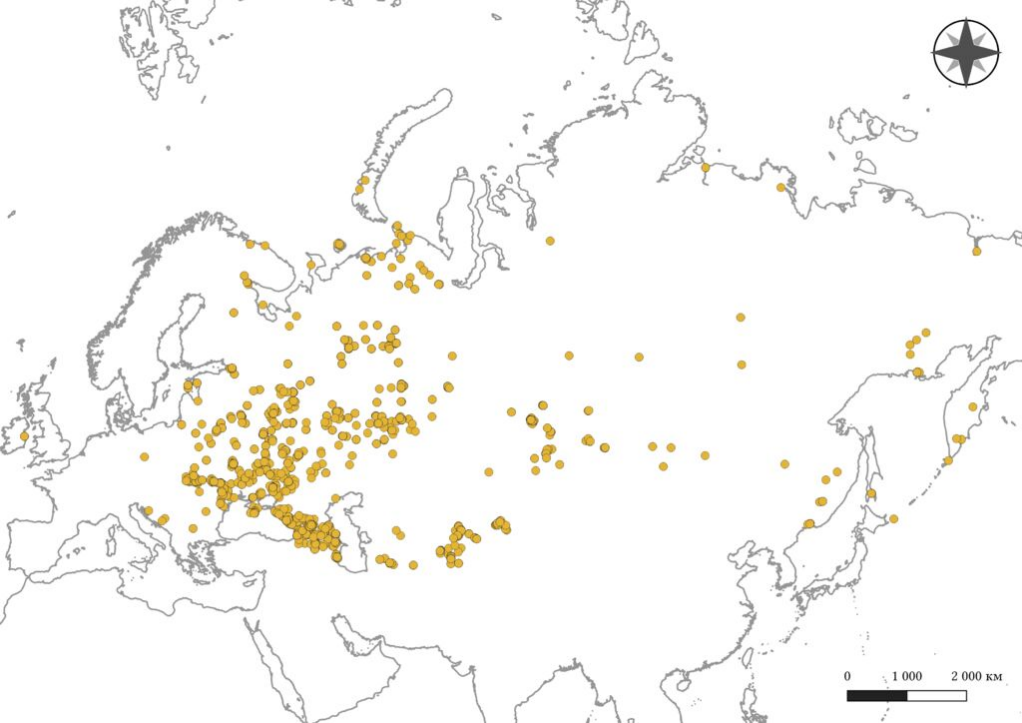
Spatial distribution of digitised earthworm occurrences.

Digitised data cover the territory of 27 countries (Fig. 4). Amongst them were: Russian Federation (3,671 occurrences), Ukraine (491), Moldova, Republic of (239), Kazakhstan (183), Georgia (129), Belarus (96), Armenia (82), Azerbaijan (79), Tajikistan (71), Kyrgyzstan (59), Uzbekistan (55), Estonia (42), Turkmenistan (34), Ireland (12), Mongolia (5), Türkiye (4), Romania (2), Latvia (2), Bulgaria (2), Bosnia and Herzegovina (2), Slovakia (1), Lithuania (1), Iran (Islamic Republic of) (1), Hungary (1), Greece (1), Germany (1), and Croatia (1). We were unable to specify a country for 44 occurrences, most of which were recorded for the Caucasus and Central Asia during the Soviet Union and earlier in the Russian Empire. The textual description of location for these occurrences is too general to select a specific country that emerged after the collapse of the Soviet Union.

### Coordinates

37.61761 and 73.49876 Latitude; −6.27701 and 165.0 Longitude.

## Taxonomic coverage

### Description

As a taxonomical basis, we used the GBIF Backbone Taxonomy and the actual checklist of earthworms (Misirlioǧlu et al. 2023), with priority given to the second source. The list of unique taxonomic names, according to the GBIF Backbone Taxonomy, accounts for 224 entries. Amongst them are 36 subspecies names (25 are accepted) and 183 species scientific names (108 are accepted and 75 are synonyms according to the GBIF Backbone Taxonomy). According to the actual checklist (Misirlioǧlu et al. 2023) that we consider to be modern systematics, the overall taxonomic scope is 109 species and 14 subspecies. Higher taxons include 23 genera belonging to five families: Acanthodrilidae (2 genera), Criodrilidae (1), Megascolecidae (2), Moniligastridae (1), and Lumbricidae (17).

Some earthworm occurrences were identified only at the generic level: *Eisenia* Malm, 1877 (2 records), *Lumbricus* Linnaeus (31), 1758, *Octolasion* Örley, 1885 (2), *Pheretima* Kinberg, 1866 (1), and even at the family level: Lumbricidae (163 records).

### Taxa included

**Table:** 

Rank	Scientific Name	
family	Acanthodrilidae	
genus	*Acanthodrilus* Perrier, 1872	
genus	*Microscolex* Rosa, 1887	
family	Criodrilidae	
genus	*Criodrilus* Hoffmeister, 1845	
family	Megascolecidae	
genus	*Amynthas* Kinberg, 1867	
genus	*Pheretima* Kinberg, 1867	
family	Moniligastridae	
genus	*Drawida* Michaelsen, 1900	
family	Lumbricidae	
genus	*Allolobophora* Eisen, 1874	
genus	*Aporrectodea* Orley, 1885	
genus	*Bimastos* Moore, 1893	
genus	*Cernosvitovia* Omodeo, 1956	
genus	*Dendrobaena* Eisen, 1874	
genus	*Dendrodriloides* Kvavadze, 2000	
genus	*Eisenia* Malm, 1877	
genus	*Eiseniella* Michaelsen, 1900	
genus	*Helodrilus* Hoffmeister, 1845	
genus	*Imetescolex* Szederjesi, Marchán, Csuzdi In Szederjesi, Marchán, Csuzdi, Sarbu, Pavlíček, Krízsik, Martin & Domínguez, 2022	
genus	*Lumbricus* Linnaeus, 1758	
genus	*Octodrilus* Omodeo, 1956	
genus	*Octolasion* Örley, 1885	
genus	*Perelia* Easton, 1983	
genus	*Proctodrilus* Zicsi, 1985	
genus	*Riphaeodrilus* Csuzdi & Pavlicek, 2005	
genus	*Scherotheca* Bouché, 1972	

## Traits coverage

### Data coverage of traits

A total of 2,437 occurrences have information about individual density, and 368 occurrences have information about individual counts. For 189 occurrences, data about the life stage of individuals are available. One hundred and five were recorded as "juvenile", most of them with identification at the family level: Lumbricidae, and 23 of them *Aporrectodeacaliginosa* (Savigny, 1826). Thirteen records as "cocoons": Lumbricidae, *Eiseniafetida* (Savigny, 1826), and *Lumbricusrubellus* Hoffmeister, 1843. Four records as "subadults". Sixty-seven occurrences were recorded as "adult", although it should be noted that this was explicitly written in the literature source. Most likely that, for the vast majority of the remaining records, these would also be "adult", otherwise it would be difficult to identify them at the species level, but this was not explicitly specified. Amongst the records for which the age stage is not specified, there are 80 entries with identification at the family level and 34 at the genus level. It is obvious that some portion of these records were based on juvenile individuals, but this was also not specified in the source literature.

## Temporal coverage

### Notes

**Figure 5.**
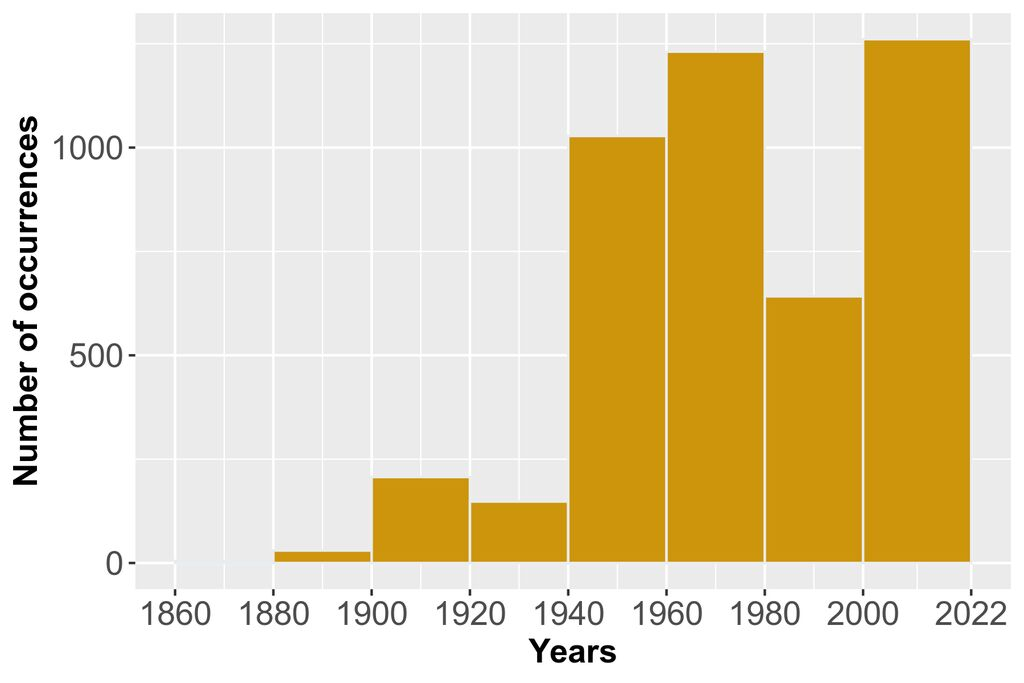
Temporal coverage for digitised earthworm occurrences.

**Figure 6.**
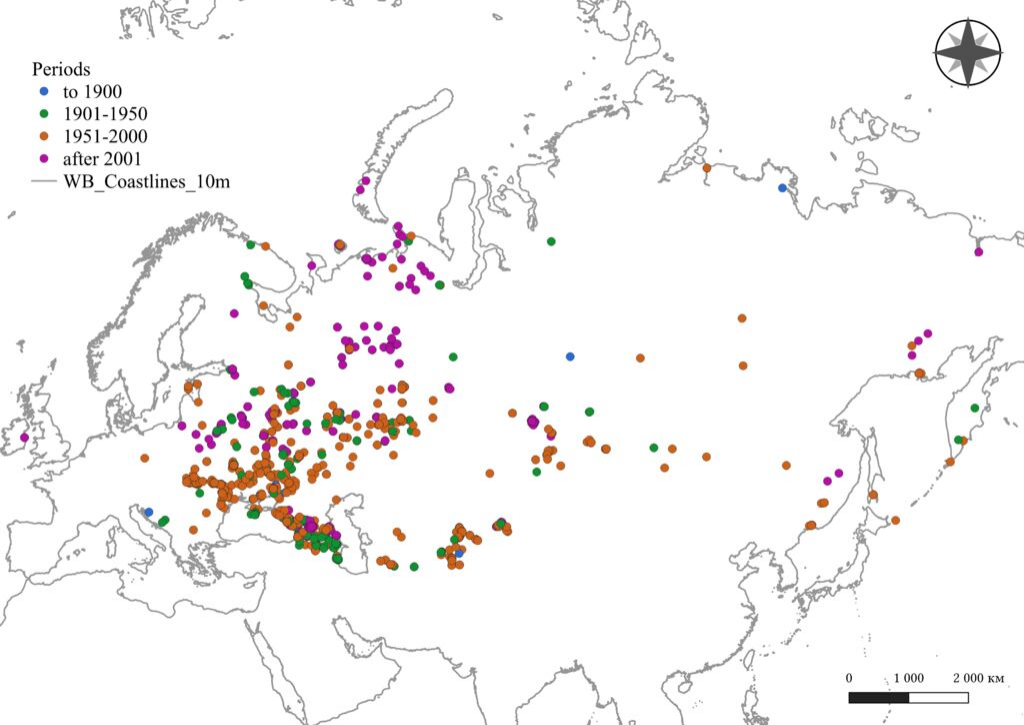
Temporal coverage for georeferenced digitised earthworm occurrences.

Our earthworm occurrence data include studies done in the 19^th^, 20^th^, and 21^st^ centuries and cover the period from 1868 to 2022, more than 150 years. Somewhat more than half of the data compiled in the dataset were collected between 1951 and 2000 (Fig. 5, Fig. 6) – 2,804 occurrences (52.9%), while for a previous, longer period of 83 years (before 1951), the volume of data amounted to only 13.6%, or 721 occurrences. Taking into account that the modern period (after 2000) has yielded almost a third of the total volume of occurrences we have consolidated, the data flow on this group is increasing. Furthermore, this volume can be greater, as we assume that we could not find all volumes of grey literature, publication of which apparently increased significantly over the previous 20–30 years.

## Usage licence

### Usage licence

Other

### IP rights notes

This work is licensed under a Creative Commons Attribution (CC-BY 4.0) License.

## Data resources

### Data package title

Earthworm occurrences from Russian-language literature

### Resource link

https://www.gbif.org/dataset/9ceef4b3-ecac-4f8a-9cca-b4a7953640ba

### Alternative identifiers

http://gbif.ru:8080/ipt/resource?r=worms2023

### Number of data sets

1

### Data set 1.

#### Data set name

Earthworm occurrences from Russian-language literature

#### Data format

Darwin Core

#### Character set

UTF-8

#### Download URL

http://gbif.ru:8080/ipt/archive.do?r=worms2023&v=1.19

#### Description

This dataset is part of the project "Quantifying the factors limiting the distribution of earthworms in European Russia: a model approach" founded by the Russian Science Foundation (23-24-00112, https://rscf.ru/en/project/23-24-00112/).

**Data set 1.** 

Column label	Column description
occurrenceID	An identifier for the dcterms:Occurrence, example: "worms:00001", "worms:05311".
basisOfRecord	The specific nature of data record. In the dataset "HumanObservation" only is used.
language	A language of the record. In the dataset "ru | en" for each occurrence.
occurrenceStatus	A statement about the presence or absence of a dcterms:Taxon at a dcterms:Location. Both "present" and "absent" are used in the dataset, there are 14 "absent" amongst them.
recordedBy	A person, group, or organisation responsible for recording the original occurrence.
eventID	An identifier for the set of information associated with a event (something that occurs at a place and time). Some occurrence in the dataset has eventID, although most records in the dataset represented results of faunistic collections (when the eventID fiels is specified, the record also has the samplingProtocol value).
countryCode	The standard code for the country in which the dcterms:Location occurs.
country	The name of the country or major administrative unit in which the dcterms:Location occurs.
stateProvince	The name of the next smaller administrative region than country (state, province, canton, department, region etc.) in which the dcterms:Location occurs.
locality	The specific description of the place.
verbatimLocality	The original textual description of the place.
identificationRemarks	Comments or notes about the dcterms:Identification. Here used as field for initial identification according to the source literature.
scientificName	The full scientific name according to the GBIF Backbone Taxonomy.
taxonID	An identifier for the set of dcterms:Taxon information. It may be a global unique identifier or an identifier specific to the dataset. According to the GBIF Backbone Taxonomy.
taxonomicStatus	The status of the use of the dwc:scientificName as a label for a taxon. Requires taxonomic opinion to define the scope of a dcterms:Taxon. Rules of priority then are used to define the taxonomic status of the nomenclature contained in that scope, combined with the experts' opinion. It must be linked to a specific taxonomic reference that defines the concept. According to the GBIF Backbone Taxonomy.
taxonRank	The taxonomic rank of the most specific name in the dwc:scientificName. According to the GBIF Backbone Taxonomy.
acceptedNameUsage	The full name, with authorship and date information if known, of the currently valid (zoological) or accepted (botanical) dcterms:Taxon. According to the actual Megadrili checklist.
acceptedNameUsageID	An identifier for the name usage (documented meaning of the name according to a source) of the direct, most proximate higher-rank parent taxon (in a classification) of the most specific element of the dwc:scientificName. Taxon key of scientific name from the Megadrili checklist according the GBIF Backbone Taxonomy.
taxonRemarks	Comments or notes about the dcterms:Taxon or name.
kingdom	The full scientific name of the kingdom in which the dcterms:Taxon is classified.
phylum	The full scientific name of the phylum or division in which the dcterms:Taxon is classified.
class	The full scientific name of the class in which the dcterms:Taxon is classified.
order	The full scientific name of the order in which the dcterms:Taxon is classified.
family	The full scientific name of the family in which the dcterms:Taxon is classified.
genus	The full scientific name of the genus in which the dcterms:Taxon is classified.
identifiedBy	A list (concatenated and separated) of names of people, groups, or organisations who assigned the dcterms:Taxon to the subject.
decimalLatitude	The geographic latitude (in decimal degrees, using the spatial reference system given in dwc:geodeticDatum) of the geographic centre of a dcterms:Location. Positive values are north of the Equator, negative values are south of it. Legal values lie between −90 and 90, inclusive.
decimalLongitude	The geographic longitude (in decimal degrees, using the spatial reference system given in dwc:geodeticDatum) of the geographic centre of a dcterms:Location. Positive values are east of the Greenwich Meridian, negative values are west of it. Legal values lie between −180 and 180, inclusive.
geodeticDatum	The ellipsoid, geodetic datum, or spatial reference system (SRS), upon which the geographic coordinates given in dwc:decimalLatitude and dwc:decimalLongitude are based. "WGS84", when it specified.
coordinateUncertaintyInMeters	The horizontal distance (in metres) from the given dwc:decimalLatitude and dwc:decimalLongitude describing the smallest circle containing the whole of the dcterms:Location. Estimated for all georeferenced occurrences.
coordinatePrecision	A decimal representation of the precision of the coordinates given in the dwc:decimalLatitude and dwc:decimalLongitude.
minimumElevationInMeters	The lower limit of the range of elevation (altitude) above sea level, in metres.
maximumElevationInMeters	The upper limit of the range of elevation (altitude) above sea level, in metres.
verbatimCoordinates	The verbatim original spatial coordinates of the dcterms:Location.
georeferencedBy	A list (concatenated and separated) of names of people, groups, or organisations who determined the georeference (spatial representation) for the dcterms:Location.
georeferencedDate	The date on which the dcterms:Location was georeferenced.
locationAccordingTo	Information about the source of this dcterms:Location information. Could be a publication (gazetteer), institution, or team of individuals.
georeferenceRemarks	Notes or comments about the spatial description determination, explaining assumptions made in addition or opposition to the those formalised in the method referred to in dwc:georeferenceProtocol.
eventDate	The date-time or interval during which a dcterms:Event occurred. For occurrences, this is the date-time when the dcterms:Event was recorded.
year	The four-digit year in which the dcterms:Event occurred, according to the Common Era Calendar.
month	The integer month in which the dcterms:Event occurred.
day	The integer day of the month on which the dcterms:Event occurred.
verbatimEventDate	The verbatim original representation of the date and time information for a dcterms:Event.
minimumDepthInMeters	The lesser depth of a range of depth below the local surface, in metres.
maximumDepthInMeters	The greater depth of a range of depth below the local surface, in metres.
habitat	A category or description of the habitat in which the dcterms:Event occurred.
individualCount	The number of individuals present at the time of the dcterms:Occurrence.
organismQuantity	A number or enumeration value for the quantity of dcterms:Organisms.
organismQuantityType	The type of quantification system used for the quantity of dcterms:Organisms.
lifeStage	The age class or life stage of the dwc:Organism(s) at the time the dcterms:Occurrence was recorded.
samplingProtocol	The names of, references to, or descriptions of the methods or protocols used during a dcterms:Event.
eventRemarks	Comments or notes about the dcterms:Event.
occurrenceRemarks	Comments or notes about the dcterms:Occurrence.
associatedReferences	A list (concatenated and separated) of identifiers (publication, bibliographic reference, global unique identifier, URI) of literature associated with the dcterms:Occurrence.

## Figures and Tables

**Table 1.** Sources included in the dataset

Publication type	Number of publications
Journal articles (peer-reviewed)	135
Monographs	4
PhD thesis	1
Proceedings	5
Conference abstracts	14
